# Retention of a depigmented eyelash in the anterior chamber for 28 years

**DOI:** 10.5935/0004-2749.2022-0105

**Published:** 2024-08-30

**Authors:** Bangtao Yao, Bin Pang

**Affiliations:** 1 Department of Ophthalmology, Nanjing Lishui People’s Hospital, Zhongda Hospital Lishui Branch, Southeast University, Nanjing, Jiangsu Province, China; 2 Department of Ophthalmology, Xuzhou Third People’s Hospital, Xuzhou Hospital Affliated to Jiangsu University Xuzhou, Jiangsu Province, China

A 52-year-old Chinese woman presented with painless blurred vision in her left eye, which she had been suffering from for decades. Her best-corrected visual acuity was 20/20 in the right eye and 20/60 in the left eye. Slit lamp examination revealed a temporal corneal scar and anterior synechia. Interestingly, a white semitransparent linear foreign body in the anterior chamber indicating an eyelash was noted (Figure, red arrow). Aqueous humor reaction was absent, and the pupils were round and light-reactive. Fundus was unremarkable. Both eyes had normal intraocular pressure. The patient had a traumatic history due to a beer bottle explosion 28 years ago, which caused an ocular injury that was sutured without the use of a microscope. After being given a sufficiently thorough explanation of the procedure at this visit, she refused to remove the eyelash. Intraocular eyelashes can result in severe intrao cular inflammation. However, depigmentation of the intraocular eyelash in the anterior chamber without inflammation for approximately 28 years, as in the present case, is rare. To the best of our knowledge, the decision to remove the intraocular eyelash is controversial.

**Figure 1:**
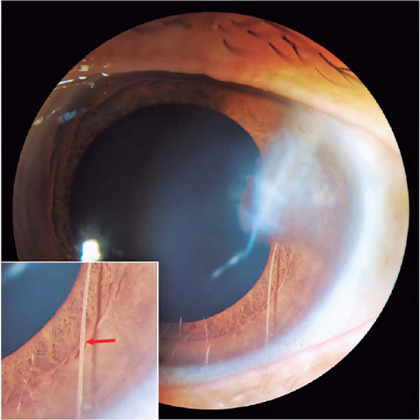
Slit lamp examination revealed a temporal corneal scar and a white semi-transparent linear foreign body in the anterior chamber, indicating an eyelash with a thin upper end embedded in the cornea and a thick lower end entering into the anterior chamber angle.
